# Associations Between Body Mass Index and Clinical Outcomes Stratified by Age in Patients Undergoing Maintenance Hemodialysis

**DOI:** 10.1111/hdi.70081

**Published:** 2026-05-15

**Authors:** Seok Hui Kang, So Young Park, Yu Jeong Lim, Bo Yeon Kim, Ji Young Choi, Jun Young Do

**Affiliations:** ^1^ Division of Nephrology, Department of Internal Medicine, College of Medicine Yeungnam University Daegu Republic of Korea; ^2^ Department of Physiology, College of Medicine Yeungnam University Daegu Republic of Korea; ^3^ Health Insurance Review and Assessment Service Wonju Republic of Korea

**Keywords:** body mass index, elderly, hemodialysis, mortality

## Abstract

**Background:**

Our study aimed to investigate the association between body mass index and clinical outcomes by age group in a population‐based cohort of patients having HD.

**Methods:**

The data collected by the Health Insurance Review and Assessment Service during HD quality assessment programs and the claims and death data were retrospectively analyzed (*n* = 71,382). The patients were divided into four groups according to their age. Each group was further divided into four groups in accordance with previous guidelines: underweight group; normal group; overweight group; and obese group.

**Results:**

The age groups were composed of the following: 41,395 patients aged < 65 years, 18,329 patients aged 65–74 years, 10,354 patients aged 75–84 years, and 1304 patients aged ≥ 85 years. In all age groups, the all‐cause mortality of the underweight group was higher than that of the normal group. Multivariable Cox regression analyses revealed that the cardiovascular event outcomes of the patients aged < 65 years and those aged 65–74 years in the overweight and obese groups were better than those in the normal group. However, in patients aged ≥ 75 years, the association between body mass index and cardiovascular event outcomes was attenuated.

**Conclusions:**

Low body mass index was associated with poor patient survival irrespective of age. Conversely, patients in overweight or obese groups had modestly lower or comparable all‐cause mortality relative to those with normal body mass index. In patients aged *<* 65 years, patients with low body mass index showed a higher risk of cardiovascular event compared with those with normal body mass index.

## 1 Introduction

Hemodialysis (HD) is the most common modality for patients with end‐stage kidney disease requiring renal replacement therapy. Patients undergoing HD experience substantially higher rates of adverse outcomes, including all‐cause mortality and cardiovascular disease, than nondialysis individuals do. Numerous studies have investigated traditional and nontraditional risk factors associated with these poor outcomes [1]. Although emerging evidence has highlighted novel biomarkers and nontraditional risk factors, traditional risk factors remain important predictors of mortality and cardiovascular disease in the general population and patients undergoing HD. However, the relationship between traditional risk factors and adverse outcomes in patients undergoing HD may be more complex than that in the general population [2]. In some cases, these factors are paradoxically related to neutral or even favorable outcomes in these patients, indicating that such risk factors may not be directly applicable in the same way as in nondialysis populations [3]. Furthermore, age likely modifies these associations; that is, traditional risk factors that are detrimental in younger patients may not exhibit the same adverse effects in elderly individuals [4, 5].

Body mass index is a well‐established indicator of obesity and important risk factor for adverse outcomes in the general population [6]. However, its interpretation may differ in specific populations such as patients undergoing HD and older adults. Previous studies demonstrated a phenomenon of reverse epidemiology in patients undergoing HD; in these patients, a low body mass index is associated with high mortality, whereas high body mass index is related to neutral or even favorable outcomes [2]. Similarly, a meta‐analysis conducted on the general elderly population has shown trends comparable with those observed in patients undergoing HD [4]. Despite extensive investigation of the relationship between body mass index and clinical outcomes in patients undergoing HD, important knowledge gaps remain. Most prior studies have evaluated body mass index as a secondary covariate among multiple prognostic factors or have relied on broad age categories (e.g., ≥ 65 or ≥ 75 years), which may obscure meaningful age‐specific heterogeneity. In particular, evidence regarding very elderly patients aged ≥ 85 years is scarce, largely due to limited sample sizes and insufficient age stratification. Moreover, many studies have primarily focused on all‐cause mortality without concurrently evaluating cardiovascular events, limiting insight into whether the prognostic significance of body mass index differs between mortality and cardiovascular risk across age groups. To address these gaps, we conducted a nationwide, population‐based study in which body mass index was analyzed as a primary exposure using both categorical and continuous approaches, with detailed age stratification, including a distinct subgroup of patients aged ≥ 85 years. By simultaneously evaluating long‐term all‐cause mortality and cardiovascular events with minimal censoring, our study aims to provide a more nuanced understanding of how the prognostic impact of body mass index varies across age groups in contemporary HD populations.

## 2 Materials and Methods

### 2.1 Data Source and Study Population

In the Republic of Korea, regular HD quality assessment programs are conducted to ensure quality control [7]. The fourth (July and December 2013), fifth (July and December 2015), sixth (March and August 2018), and seventh (October 2020 and March 2021) HD quality assessment programs were conducted for adult patients (age ≥ 18 years) who have been receiving maintenance HD for at least 3 months and at least twice per week. The data collected by the Health Insurance Review and Assessment Service during HD quality assessment programs and the claims and death data were retrospectively analyzed. From an initial pool of 127,302 participants in the fourth–seventh HD quality assessment programs, only the first of multiple participants (*n* = 53,738 with repeated inclusions) were included; the participants who underwent HD with a catheter (*n* = 1763) and those with insufficient data (*n* = 271), without body mass index assessments (*n* = 14), and with extreme body mass index (≤ 0.1 or 99.9th ≥ percentile) were excluded. Thus, our study cohort had 71,382 patients. The research protocol was approved by the Institutional Review Board of Yeungnam University Medical Center (approval no. YUMC 2023‐12‐012). Informed consent was not required because patient records and information were anonymized and de‐identified before the analysis.

### 2.2 Exposure

Body mass index was calculated using the post‐HD body weight per height square (kg/m^2^). During HD quality assessment programs, body mass index data were collected for 6 months, and the average of the assessments collected monthly was used. The patients were divided into four groups according to their age; < 65, 65–74, 75–84, or ≥ 85 years. Each group was further divided into four groups in accordance with previous guidelines: underweight group, body mass index < 18.5 kg/m^2^; normal group, 18.5 kg/m^2^ ≤ body mass index ≤ 24.9 kg/m^2^; overweight group, 25.0 kg/m^2^ ≤ body mass index ≤ 29.9 kg/m^2^; and obese group, body mass index ≥ 30 kg/m^2^ [6].

### 2.3 Study Variables

Data on several variables, such as age, sex, HD vintage (months), underlying etiology of end‐stage kidney disease, and vascular access type used, were complied. The following clinical parameters were documented during the evaluation: blood hemoglobin levels (g/dL); serum levels of albumin (g/dL), calcium (mg/dL), phosphorus (mg/dL), and creatinine (mg/dL); Kt/V_urea_; and ultrafiltration volume (L/session). They were collected monthly, and all laboratory values were derived as averages from these monthly datasets. Kt/V_urea_ was calculated using the Daugirdas equation [8].

Medications such as renin–angiotensin system blockers, aspirin, clopidogrel, and statins were evaluated using the medication codes provided in Table S1. Their use was defined as one or more prescriptions identified during an HD quality assessment program. Before HD quality assessments were conducted, comorbidities were evaluated for 1 year and defined using the Charlson comorbidity index, which encompasses 17 different comorbid conditions [9, 10, 11]. Charlson comorbidity index scores were computed for all patients. The presence of myocardial infarction or congestive heart failure was identified on the basis of ICD‐10 codes.

### 2.4 Outcomes

The patients were followed up until June 2024. All‐cause mortality was evaluated as the primary outcome, and cardiovascular events were examined as the secondary outcome. The incidence of death and death date were determined from the Health Insurance Review and Assessment Service. The incidence of cardiovascular events including myocardial infarction, stroke, and revascularization regardless of survival or death, was evaluated as previously described [12]. Patients with cardiovascular event within 1 year before the assessment and 6 months of each assessment were excluded in cardiovascular event analyses. If a patient was transferred to peritoneal dialysis or kidney transplantation without an event, it was considered censored at that time.

### 2.5 Statistical Analyses

Data were analyzed using two statistical software packages: SAS Enterprise Guide v7.1 and R v3.5.1. Categorical variables were presented as frequencies and percentages, while continuous variables were presented as means and standard deviations. The statistical significance of differences between categorical variables was examined using Pearson's *χ* ^2^ test or Fisher's exact test. Differences between continuous variables were assessed via one‐way analysis of variance followed by Tukey's post hoc test.

Survival curves were estimated using Kaplan–Meier curves. The *p*‐values for their comparison were determined using the log‐rank test. Hazard ratio (HR) and confidence interval (CI) were calculated via Cox regression analyses. Multivariable Cox regression analyses were adjusted for age; sex; vascular access type; Charlson comorbidity index score; HD vintage; ultrafiltration volume; Kt/V_urea_; blood levels of hemoglobin, albumin, creatinine, phosphorus, and calcium; use of renin–angiotensin system blockers, aspirin, clopidogrel, or statins; and myocardial infarction or congestive heart failure. Multivariable Cox regression analyses were performed using the enter mode. Restricted cubic spline models adjusted for the same covariates included in the multivariable Cox models were applied to explore the nonlinear associations between body mass index and outcomes. Data were considered statistically significant at *p* < 0.05.

## 3 Results

### 3.1 Baseline Characteristics

The age groups were composed of the following: 41,395 patients aged < 65 years, 18,329 patients aged 65–74 years, 10,354 patients aged 75–84 years, and 1304 patients aged ≥ 85 years (Table 1). The proportions of male sex, arteriovenous fistula, diabetes, and use of renin–angiotensin system blockers in the patients aged ≥ 85 years were lower than those in the other age groups; the proportions of aspirin and clopidogrel use and prevalence of myocardial infarction or congestive heart failure were higher in the former than in the latter. Moreover, the levels of Kt/V_urea_ of the patients aged ≥ 85 years increased compared with those of the other age groups; their levels of ultrafiltration volume, serum albumin, phosphorus, calcium, and creatinine were lower than those of the other age group. Conversely, their Charlson comorbidity index scores were higher than those of the other age groups. The underweight, normal, overweight, and obese groups had 3775, 28,073, 7851, and 1696 patients aged < 65 years, respectively; 1417, 13,104, 3382, and 426 patients aged 65–74 years, respectively; 1056, 7436, 1690, and 172 patients aged 75–84 years, respectively; and 182, 940, 169, and 13 patients aged ≥ 85 years, respectively (Table S2).

**TABLE 1:** Clinical characteristics of the patients.

	< 65 years old (*n* = 41,395)	66–74 years old (*n* = 18,329)	75–84 years old (*n* = 10,354)	≥ 85 years old (*n* = 1304)	*p*
Age (years)	52.1 ± 9.0	69.4 ± 2.9^a^	78.5 ± 2.6^a^, ^b^	87.1 ± 2.3^a^, ^b^, ^c^	< 0.001
Sex (male, %)	25,832 (62%)	10,845 (59%)	5768 (56%)	680 (52%)	< 0.001
Body mass index (kg/m^2^)	22.8 ± 3.6	22.7 ± 3.2^a^	22.3 ± 3.1^a^, ^b^	21.8 ± 3.1^a^, ^b^, ^c^	< 0.001
Hemodialysis vintage (months)	58 ± 64	48 ± 53^a^	39 ± 41^a^, ^b^	34 ± 33^a^, ^b^	< 0.001
Underlying causes of end‐stage kidney disease					< 0.001
Diabetes mellitus	17,466 (42%)	9767 (53%)	4872 (47%)	465 (36%)	
Hypertension	10,045 (24%)	4692 (26%)	3329 (32%)	532 (41%)	
Glomerulonephritis	5303 (13%)	1115 (6.1%)	522 (5.0%)	52 (4.0%)	
Others	3974 (9.6%)	1160 (6.3%)	599 (5.8%)	83 (6.4%)	
Unknown	4607 (11%)	1595 (8.7%)	1032 (10%)	172 (13%)	
Charlson Comorbidity Index score	7.1 ± 2.9	8.4 ± 3.0^a^	8.7 ± 3.0^a^, ^b^	8.9 ± 3.1^a^, ^b^	< 0.001
Arteriovenous fistula	36,611 (88%)	15,217 (83%)	8057 (78%)	922 (71%)	< 0.001
Kt/V_urea_	1.51 ± 0.25	1.53 ± 0.24^a^	1.56 ± 0.25^a^, ^b^	1.59 ± 0.25^a^, ^b^, ^c^	< 0.001
Ultrafiltration volume (L/session)	2.5 ± 0.9	2.1 ± 0.9^a^	1.9 ± 0.9^a^, ^b^	1.8 ± 0.8^a^, ^b^, ^c^	< 0.001
Hemoglobin (g/dL)	10.7 ± 0.8	10.6 ± 0.7^a^	10.6 ± 0.7^a^	10.6 ± 0.7^a^	< 0.001
Serum albumin (g/dL)	4.06 ± 0.33	3.93 ± 0.32	3.85 ± 0.32^a^, ^b^	3.77 ± 0.33^a^, ^b^, ^c^	< 0.001
Serum phosphorus (mg/dL)	5.3 ± 1.3	4.6 ± 1.1^a^	4.4 ± 1.0^a^, ^b^	4.2 ± 1.0^a^, ^b^, ^c^	< 0.001
Serum calcium (mg/dL)	8.92 ± 0.79	8.81 ± 0.75^a^	8.73 ± 0.70^a^, ^b^	8.68 ± 0.66^a^, ^b^	< 0.001
Serum creatinine (mg/dL)	10.3 ± 2.7	8.6 ± 2.3^a^	7.6 ± 2.2^a^, ^b^	7.0 ± 2.0^a^, ^b^, ^c^	< 0.001
Use of renin–angiotensin system blocker	27,319 (66%)	12,239 (67%)	6350 (61%)	722 (55%)	< 0.001
Use of aspirin	9284 (22%)	5506 (30%)	3502 (35%)	452 (35%)	< 0.001
Use of clopidogrel	4899 (12%)	3451 (19%)	2384 (23%)	311 (24%)	< 0.001
Use of statins	17,355 (42%)	9568 (52%)	5348 (52%)	638 (49%)	< 0.001
Myocardial infarction or congestive heart failure	17,470 (42%)	9524 (52%)	5921 (57%)	788 (60%)	< 0.001

*Note:* Data were presented as mean ± standard deviation for continuous variables and numbers (percentages) for categorical variables. *p*‐values were tested using one‐way analysis of variance, followed by Tukey's post hoc test and Pearson's *χ* ^2^ test for categorical variables.

^a^ *p* < 0.05 versus < 65 years of age.

^b^ *p* < 0.05 versus 66–74 years of age.

^c^ *p* < 0.05 versus 75–84 years of age.

### 3.2 Overall Survival According to Body Mass Index Groups

**FIGURE 1:**
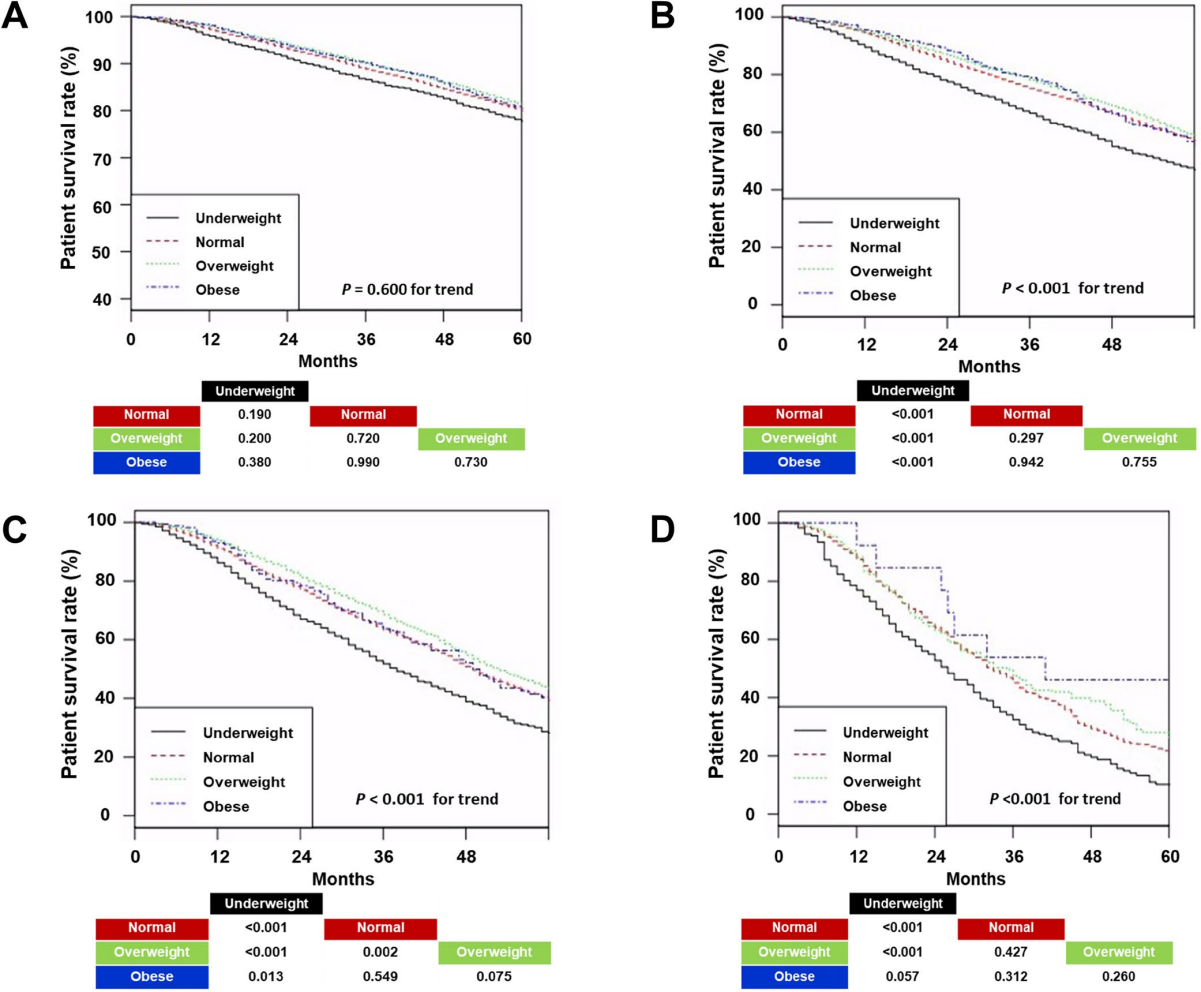
Kaplan–Meier curves of patient survival based on body mass index groups across different age categories. Survival curves of patients aged < 65 years (A), 65–74 years (B), 75–84 years (C), and ≥ 85 years (D). *p*‐values for pairwise comparisons with log‐rank tests were presented at the bottom of the graph. Underweight group, patients with body mass index < 18.5 kg/m^2^; Normal group, patients with 18.5 ≤ body mass index ≤ 24.9 kg/m^2^; Overweight group, patients with 25.0 ≤ body mass index ≤ 29.9 kg/m^2^; Obese group, patients with body mass index ≥ 30 kg/m^2^.

The follow‐up duration of the different age groups varied as follows: 71 ± 37 months in patients aged < 65 years, 58 ± 33 months in patients aged 65–74 years, 46 ± 27 months in patients aged 75–84 years, and 34 ± 21 months in patients aged ≥ 85 years. The 5‐year survival rates of the underweight, normal, overweight, and obese groups were 78%, 80%, 81%, and 81% in patients aged < 65 years, respectively; 47%, 57%, 59%, and 55% in patients aged 65–74 years, respectively; 28%, 39%, 43%, and 40% in patients aged 75–84 years, respectively; and 10%, 21%, 26%, and 46% in patients aged ≥ 85 years, respectively (Figure 1).

Among the patients aged < 65 years, the body mass index group was not associated with patient survival. However, among all age groups except the group of < 65 years old, the underweight group had the poorest patient survival. Table 2 presents the results of the Cox regression analyses. Multivariable Cox regression analyses showed that the all‐cause mortality of the underweight group was higher than that of the normal or overweight groups in all age groups. The all‐cause mortality of the obese group was similar to that of the normal group.

**TABLE 2:** Body mass index and all‐cause mortality or cardiovascular events based on age groups.

	All‐cause mortality				Cardiovascular events			
	Univariate		Multivariate		Univariate		Multivariate	
	HR (95% CI)	*p*	HR (95% CI)	*p*	HR (95% CI)	*p*	HR (95% CI)	*p*
< 65 years old								
Underweight	1.04 (0.98–1.10)	0.202	1.30 (1.22–1.38)	< 0.001	0.88 (0.81–0.95)	0.002	1.08 (0.99–1.18)	0.060
Overweight	0.99 (0.95–1.04)	0.698	0.91 (0.87–0.96)	< 0.001	1.02 (0.96–1.09)	0.434	0.93 (0.87–0.99)	0.018
Obese	1.00 (0.91–1.10)	0.970	1.01 (0.91–1.11)	0.900	0.88 (0.77–0.99)	0.043	0.84 (0.74–0.96)	0.013
65–74 years old								
Underweight	1.31 (1.23–1.40)	< 0.001	1.33 (1.24–1.42)	< 0.001	1.04 (0.92–1.17)	0.498	1.11 (0.99–1.26)	0.080
Overweight	0.97 (0.92–1.02)	0.192	0.94 (0.89–0.99)	0.014	0.89 (0.82–0.97)	0.009	0.83 (0.76–0.91)	< 0.001
Obese	1.00 (0.87–1.14)	0.951	0.96 (0.84–1.10)	0.564	0.76 (0.60–0.97)	0.030	0.69 (0.53–0.88)	0.003
75–84 years old								
Underweight	1.37 (1.28–1.47)	< 0.001	1.37 (1.27–1.48)	< 0.001	1.04 (0.89–1.22)	0.586	1.13 (0.96–1.33)	0.140
Overweight	0.90 (0.84–0.96)	< 0.001	0.90 (0.85–0.97)	0.003	0.99 (0.87–1.13)	0.907	0.95 (0.83–1.08)	0.425
Obese	1.06 (0.88–1.28)	0.545	1.09 (0.90–1.32)	0.379	1.11 (0.76–1.63)	0.587	1.03 (0.70–1.51)	0.883
≥ 85 years old								
Underweight	1.44 (1.21–1.70)	< 0.001	1.42 (1.19–1.70)	< 0.001	0.85 (0.54–1.35)	0.498	0.76 (0.46–1.25)	0.276
Overweight	0.93 (0.77–1.12)	0.429	0.84 (0.69–1.02)	0.075	1.18 (0.77–1.81)	0.437	1.06 (0.68–1.66)	0.782
Obese	0.70 (0.37–1.31)	0.263	0.66 (0.35–1.25)	0.203	—		—	

*Note:* Multivariate analysis was adjusted for age, sex, vascular access type, hemodialysis vintage, underlying cause of end‐stage kidney disease; Charlson Comorbidity Index score, Kt/Vurea, ultrafiltration volume, hemoglobin, serum albumin, serum creatinine, serum phosphorus, serum calcium, use of renin–angiotensin system blocker, statin, clopidogrel, or aspirin, and presence myocardial infarction or congestive heart failure; this analysis was performed using the enter mode. Ref group: patients with 18.5 kg/m^2^ ≤ body mass index ≤ 24.9 kg/m^2^.

Abbreviations: CI, confidence interval; HR, hazard ratio.

### 3.3 Cardiovascular Event‐Free Survival According to Body Mass Index Groups

**FIGURE 2:**
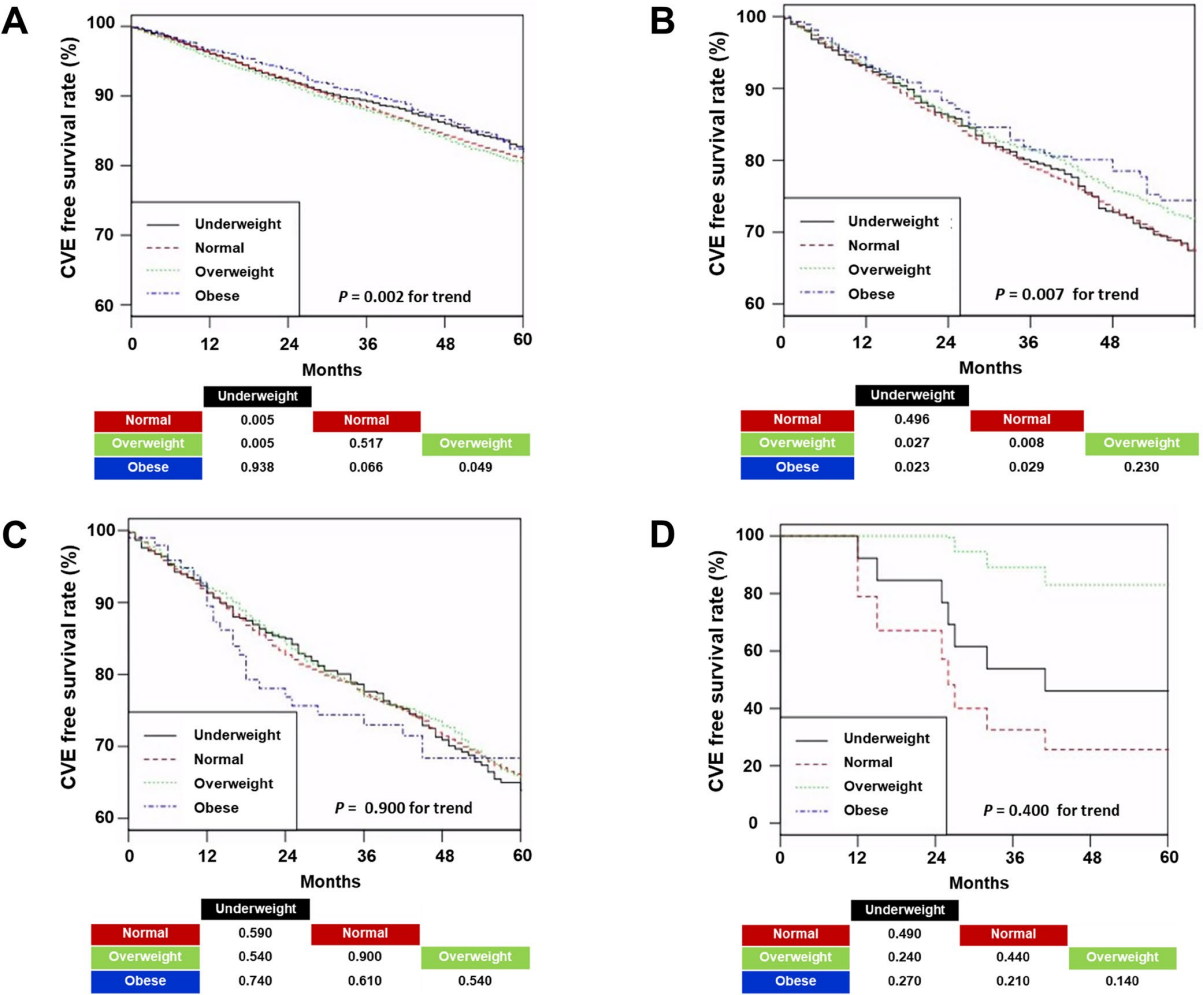
Kaplan–Meier curves of cardiovascular events based on body mass index groups across different age categories. Survival curves of patients in the following age groups: < 65 years (A), 65–74 years (B), 75–84 years (C), and ≥ 85 years (D). *p*‐values for pairwise comparisons with log‐rank tests were presented at the bottom of the graph. Underweight group, patients with body mass index < 18.5 kg/m^2^; Normal group, patients with 18.5 ≤ body mass index ≤ 24.9 kg/m^2^; Overweight group, patients with 25.0 ≤ body mass index ≤ 29.9 kg/m^2^; Obese group, patients with body mass index ≥ 30 kg/m^2^.

The 5‐year cardiovascular event‐free rates of the underweight, normal, overweight, and obese groups were 82%, 81%, 80%, and 82% in patients aged < 65 years, respectively; 67%, 67%, 72%, and 74% in patients aged 65–74 years, respectively; and 64%, 66%, 66%, and 68% in patients aged 75–84 years, respectively (Figure 2). Those of the underweight, normal, and overweight groups were 68%, 67%, and 53% in patients aged ≥ 85 years, respectively. All patients aged ≥ 85 in the obese group had a prevalent cardiovascular event, and cardiovascular event‐free survival analyses were not performed in this group. Multivariable Cox regression analyses showed that the cardiovascular event outcomes of the patients aged < 65 years or 65–74 years in the overweight or obese groups were better than those of the normal group.

### 3.4 Spline Curves Between Body Mass Index and Clinical Outcomes

**FIGURE 3:**
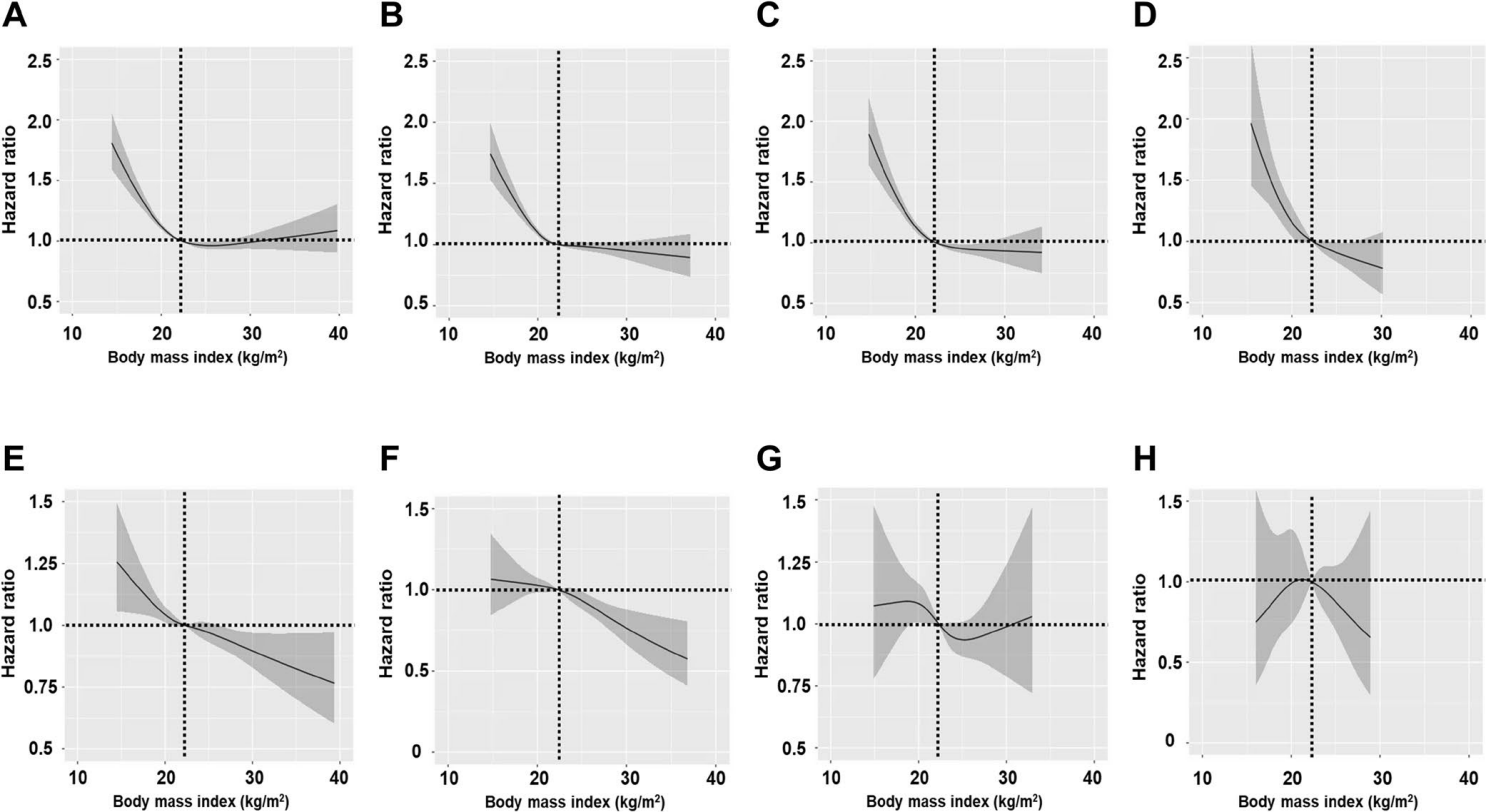
Spline curves illustrating the hazard ratios and 95% confidence intervals of clinical outcomes according to body mass index (A–D, all‐cause mortality; E–H, cardiovascular events). Survival curves of patients in the following age groups: < 65 years (A), 65–74 years (B), 75–84 years (C), and ≥ 85 years; cardiovascular event curves of the following age groups: < 65 years (E), 65–74 years (F), 75–84 years (G), and ≥ 85 years (H). The reference point was established at body mass index of 22.3 kg/m^2^. Data were plotted using a multivariable model, and adjustments were made in terms of the following factors: age; sex; vascular access type; hemodialysis vintage; underlying cause of end‐stage kidney disease; Charlson Comorbidity Index score; Kt/V_urea_; ultrafiltration volume; hemoglobin, serum albumin, serum creatinine, serum phosphorus, and serum calcium levels; use of renin–angiotensin system blocker, statin, clopidogrel, or aspirin; and presence of myocardial infarction or congestive heart failure.

Body mass index levels below the median (22.3 kg/m^2^) were associated with a higher all‐cause mortality than the median body mass index in all age groups (Figure 3A–D). Body mass index levels above the median were initially related to a lower all‐cause mortality than median body mass index; however, as the body mass index further increased and exceeded a specific threshold, the initially protective relationship decreased and did not reach statistical significance in all age groups.

In individuals aged < 65 years, the risk of cardiovascular event was higher when body mass index was below the median; conversely, it was lower when body mass index was above the median (Figure 3E). A similar trend was observed in age group of 65–74 years (Figure 3F). In patients aged ≥ 75 years, the body mass index did not have a clear association with the cardiovascular event risk (Figure 3G,H).

## 4 Discussion

Our study analyzed the association between body mass index and all‐cause mortality or cardiovascular event in a population‐based cohort of patients undergoing HD in South Korea. In all age groups, the all‐cause mortality of the underweight group was higher than that of the normal group. Multivariable Cox regression analyses revealed that the cardiovascular event outcomes of the patients aged < 65 years and those aged 65–74 years in the overweight and obese groups were better than those in the normal group. However, in patients aged ≥ 75 years, the association between body mass index and cardiovascular event outcomes was attenuated.

The association between body mass index and adverse outcomes in patients undergoing HD has been widely studied, and it differs from patterns observed in the general population. In such patients, a low body mass index is consistently related to increased all‐cause mortality and cardiovascular events; conversely, a high body mass index often shows a neutral or even protective effect, which is referred to as reverse epidemiology [2, 13, 14]. This phenomenon may be explained via several proposed mechanisms, including better nutritional status and greater metabolic reserves, as well as the anti‐inflammatory role of adipose tissue. Our findings were consistent with this paradigm, particularly in younger patients (< 65 years) undergoing HD, whose lower body mass index was strongly associated with higher mortality and cardiovascular event risk, and a higher body mass index exhibited a beneficial or neutral association.

Previous meta‐analyses have demonstrated relatively consistent trends regarding the association between body mass index and mortality. Herselman et al. analyzed 31 studies published between 1996 and 2008 and reported an inverse association between body mass index and mortality in 18 of these studies [15]. This inverse relationship was more frequently observed in studies enrolling patients with a mean age of 60 years or older, whereas the association was weaker or absent in studies focusing on younger populations with a mean age below 50 years. Among the seven studies that evaluated cardiovascular mortality, more than half failed to demonstrate a significant association between body mass index and cardiovascular mortality. Subsequently, two categorical body mass index‐based meta‐analyses published in 2014 and 2017 consistently reported that higher body mass index was associated with improved survival in dialysis populations [16, 17]. However, these analyses primarily evaluated overall populations or focused on monotonic body mass index categories, and detailed analyses of very elderly patients were limited.

Interestingly, our age‐stratified analysis revealed that the protective association of a high body mass index weakened progressively with advancing age. In patients aged ≥ 75 years, the relationship between body mass index and adverse outcomes became less distinct, and a high body mass index had no clear protective effect. These findings were consistent with previous studies in the general elderly population, suggesting that body mass index may be less predictive of outcomes in advanced age because of the confounding influence of frailty, sarcopenia, and chronic illness [4, 18, 19]. In elderly patients undergoing HD, weight loss may reflect underlying catabolic states or comorbidities rather than intentional weight control; as such, the interpretation of body mass index as a risk marker becomes complex. Our results highlighted the need for age‐specific approaches in evaluating nutritional and anthropometric risk in patients undergoing HD and suggested that reliance on body mass index alone might be insufficient in the very elderly. Additional metrics such as body composition, muscle mass, and functional status should be incorporated in future studies and clinical assessments. Previous studies involving elderly patients undergoing HD demonstrated that changes in body mass index, rather than a simple measurement of body mass index, can be used to predict outcomes [20, 21].

Similar to our study, a previous study analyzed the association between body mass index and all‐cause mortality by age group from data on Korean patients undergoing HD [22]. Their study was based on a national registry; however, unlike our dataset, which was derived from a mandatory quality assessment program that included almost all HD centers in Korea, their registry relies on voluntary patient enrollment and data submission by individual centers and practitioners. Thus, their dataset may have been more prone to selection bias and higher dropout rates. Similarly, Villain et al. reported that the association between body mass index and mortality is inversely linear in patients aged ≥ 75 years undergoing HD [20]. However, Polinder‐Bos et al. found that among patients aged ≥ 65 years who survived at least 1 year, a low body mass index is related to favorable long‐term survival; this result suggests that the prognostic value of body mass index may vary with time and patient frailty status [23]. Our study utilized a more comprehensive dataset that included dialysis‐related laboratory data and insurance claims information, enhancing its reliability. We included a distinct subgroup of patients aged ≥ 85 years, enrolled a relatively larger number of patients aged ≥ 65 years, and analyzed patients from the 2013 program onward, which reflected a more contemporary HD population. Our median follow‐up duration (3–5 years) was also longer than the approximately 2‐year follow‐up in a previous study involving national registry data [23]. Moreover, we analyzed cardiovascular events in addition to mortality and observed that a high body mass index was associated with a low cardiovascular event risk in patients under 75 years of age. The weaker association between body mass index and cardiovascular events in our elderly group might be explained by the exclusion of a substantial number of older patients with preexisting cardiovascular events from the cardiovascular event analysis and by the relatively smaller number of elderly patients; thus, these factors likely reduced the statistical power to detect significant associations.

In patients aged ≥ 75 years, the associations between body mass index and clinical outcomes were attenuated. These findings should be interpreted cautiously and not overstated. First, the reduced sample size in older subgroups may have limited statistical power, thereby reducing the ability to detect modest associations. Second, in older patients undergoing HD, body mass index may be a less reliable surrogate for nutritional status or metabolic reserve due to the increasing prevalence of frailty, sarcopenia, and multiple comorbidities. The relatively high burden of comorbidities may have contributed to frailty independent of body mass index. In this context, a higher body mass index may not necessarily reflect greater lean body mass or physiological resilience. Finally, the burden of comorbid conditions and competing risks of noncardiovascular mortality in older patients may diminish the relative contribution of body mass index to clinical outcomes. Together, these factors may partially explain the attenuated associations observed in older age groups.

Two studies using Swedish registry data and data from a Japanese center reported a higher mortality risk among patients with a body mass index < 18.5 kg/m^2^ and a lower mortality risk among those with higher body mass index values compared with the reference group [24, 25]. Similarly, Kitajima et al. analyzed data from 132 HD patients at a Japanese center and divided participants into two groups based on a body mass index threshold of 18 kg/m^2^, demonstrating an increased mortality risk in patients with low body mass index [26]. Barra et al., using Brazilian registry data, examined body mass index as a continuous variable and its association with all‐cause mortality [27]. Their findings likewise showed a decreasing mortality risk with increasing body mass index. While these studies consistently demonstrated an inverse association between body mass index and mortality in HD patients, none performed age‐stratified analyses, which may provide additional insight into potential effect modification.

In more recent studies, body mass index has often been identified as one of several prognostic factors rather than being analyzed as a primary exposure of interest. Oliva et al. analyzed 704 HD patients aged 75 years or older and demonstrated an association between low body mass index (< 20 kg/m^2^) and increased mortality in this very elderly population [28]. Nagai et al. evaluated 314 HD patients aged 65–84 years and reported an inverse association between body mass index and all‐cause mortality using Cox regression, with body mass index treated as a continuous variable [29]. Similarly, Park et al. analyzed Korean registry data with patients stratified into age groups of < 65, 65–74, and ≥ 75 years and reported outcomes over a median follow‐up of approximately 20–23 months [30]. In that analysis, body mass index was included as a covariate among multiple risk factors, and each 1 kg/m^2^ increase in body mass index was associated with an approximately 5% reduction in mortality risk. Collectively, these studies indicate that even in older HD populations, body mass index has primarily been evaluated as a secondary covariate rather than as a primary exposure. Moreover, studies specifically focusing on very elderly patients and using well‐categorized body mass index as the primary factor to assess all‐cause mortality remain limited, underscoring the need for further investigation in this population.

Although the association between body mass index and clinical outcomes in patients undergoing HD has been reported previously, we believe that our study provides several important and distinct contributions that advance existing knowledge. First, unlike prior studies that broadly categorized elderly patients (e.g., ≥ 65 or ≥ 75 years), we further stratified older patients and analyzed a distinct subgroup of very elderly individuals aged ≥ 85 years. This approach allowed us to demonstrate that the protective association between higher body mass index and cardiovascular outcomes is markedly attenuated in this age group. To our knowledge, this finding has not been clearly established in previous studies, largely due to the limited sample size of very elderly patients undergoing HD or the use of a wide range of ages. Second, body mass index was analyzed as a primary prognostic factor, rather than a secondary covariate, allowing a more focused assessment of its clinical relevance. We evaluated body mass index using both categorical approaches (four predefined body mass index categories) and as a continuous variable using restricted cubic spline models. This comprehensive analytic strategy enabled us to capture nonlinear associations and move beyond dichotomous or monotonic trend‐based interpretations commonly used in earlier studies.

Third, our analysis was based on data from a mandatory nationwide HD quality assessment program that includes nearly all dialysis centers in Korea, thereby minimizing selection bias compared with voluntary registries that are prone to substantial loss to follow‐up. In Korea, patients undergoing HD are registered as individuals with disabilities and receive full medical cost coverage through the National Health Insurance system. Consequently, the Health Insurance Review and Assessment Service continuously monitors HD‐related medical claims and manages claim discontinuation, including those occurring immediately after death, with a high degree of accuracy. Because mortality data in our study are derived from insurance claims and reasons for claim termination, death can be ascertained very reliably during the follow‐up period, regardless of patient transfer between institutions. Therefore, this dataset allows reliable ascertainment of all‐cause mortality, with minimal censoring except for modality changes such as kidney transplantation or transition to peritoneal dialysis. In our study, the number of patients transferred to peritoneal dialysis or underwent kidney transplantation was 5363 (13%) among those aged < 65 years, 332 (1.8%) among those aged 65–74 years, 24 (0.3%) among those aged 75–84 years, and zero among those aged in ≥ 85 years. Consequently, our study benefits from long‐term follow‐up and a relatively low risk of informative censoring. Fourth, we simultaneously evaluated both all‐cause mortality and cardiovascular events as clinical outcomes. This dual‐outcome approach allowed us to demonstrate that the age‐dependent modification of the body mass index–outcome relationship differs between mortality and cardiovascular risk, thereby providing new insights into the metabolic and cardiovascular implications of body mass index in older patients undergoing HD. Overall, by incorporating finer age stratification in an aging dialysis population, treating body mass index as a primary exposure using both categorical and continuous analyses, and leveraging a contemporary nationwide cohort with long‐term follow‐up and low censoring, our study offers a more nuanced understanding of how the prognostic significance of body mass index varies with age in patients undergoing HD.

Our study has several limitations. First, this was a retrospective observational study that analyzed data of prevalent patients undergoing HD. Consequently, drawing clear conclusions about the causal relationship between the two variables is challenging. The use of prevalent cohorts may introduce selection bias, as baseline characteristics differed across body mass index categories despite multivariable adjustment. Therefore, residual confounding cannot be fully excluded, and the results should be interpreted as associative rather than causal. Second, body mass index measurements were not obtained using a standardized protocol across centers. Height and weight assessment were measured as part of routine clinical practice, and the timing of body weight assessment, as well as measurement equipment, may have varied between institutions. The lack of uniform protocols for height and weight assessment across institutions may have resulted in inter‐center variability and nondifferential misclassification of body mass index, which has attenuated the observed associations. Third, we relied solely on ICD‐10 diagnostic codes or procedural information to assess the effects of cardiovascular events. Therefore, misclassification of cardiovascular outcomes is possible, particularly for events that may be underreported or heterogeneously coded across institutions. Our approach may lead to over‐ or under‐ascertainment of outcomes, thereby introducing potential errors in assessing the association between body mass index and cardiovascular events. Furthermore, because outcome misclassification based on administrative codes is likely to be nondifferential with respect to body mass index categories, this limitation would be expected to attenuate observed associations rather than generate spurious findings. Consequently, the true association between body mass index and cardiovascular outcomes may be stronger than that observed in the present study. Future studies that integrate administrative data with detailed clinical assessments, including echocardiography, electrocardiography, and imaging modalities, could help address this limitation and provide a more precise evaluation of cardiovascular events. Fourth, the data in our study included only information regarding all‐cause mortality as an outcome and provided limited details on specific death causes, such as cachexia, infection, and cardiovascular diseases. The absence of cause‐specific mortality data limits our ability to distinguish whether the observed associations between body mass index and survival are driven primarily by cardiovascular, infectious, or other noncardiovascular causes. Such information would have been valuable for elucidating potential biological mechanisms underlying the relationship between body mass index and mortality in patients undergoing HD. In particular, cause‐specific mortality data could have helped clarify whether age‐dependent modification of the body mass index–mortality association differs according to specific causes of death. Moreover, data on additional outcomes, such as quality of life, could contribute to a more comprehensive evaluation of patient‐centered and subjective effects beyond direct mortality. Fifth, overall, the number of patients aged ≥ 85 years was significantly lower than that in the other age groups. Therefore, the results of multivariable analyses should be cautiously interpreted. The limited sample size in this age group may have reduced statistical power, increasing the risk of type II error and limiting our ability to detect modest associations between body mass index and clinical outcomes. Consequently, the absence or attenuation of associations observed in patients aged ≥ 85 years should not be interpreted as definitive evidence of no effect.

In conclusion, body mass index < 18.5 kg/m^2^, as defined as a low body mass index, was associated with poor patient survival irrespective of age. Conversely, patients in overweight or obese groups had modestly lower or comparable all‐cause mortality relative to those with a normal body mass index. In patients aged *<* 65 years, patients with a low body mass index showed a higher risk of cardiovascular event compared with those with a normal body mass index. These findings suggested that even in elderly populations undergoing HD, clinical efforts may focus on addressing the risks associated with underweight status rather than overweight or obesity.

## Funding

This work was supported by the Medical Research Center Program through the National Research Foundation (NRF) of Korea funded by the Ministry of Science, ICT, and Future Planning (2022R1A5A2018865), and the Basic Science Research Program through the NRF of Korea, funded by the Ministry of Education (2022R1I1A3072966).

## Ethics Statement

The study was conducted ethically in accordance with the World Medical Association Declaration of Helsinki. The study was approved by the institutional review board (IRB) of the Yeungnam University Medical Center (approval no. YUMC 2023‐12‐012).

## Consent

Informed consent was not obtained from the patients since the records and information of the participants were anonymized and de‐identified before the analysis. The IRB also waived the need for obtaining informed consent.

## Conflicts of Interest

The authors declare no conflicts of interest.

## Supporting information


**Table S1:** Medication types and health insurance review and assessment service codes.
**Table S2:** Clinical characteristics according to body mass index in patients aged ≥ 85 years.

## Data Availability

The raw data were generated by the Health Insurance Review and Assessment Service. The database can be requested from the Health Insurance Review and Assessment Service by sending a study proposal including the purpose of the study, study design, and duration of analysis through the web site (https://www.hira.or.kr). The authors cannot distribute the data without permission.
