# Supplementary material for: Associations Between Body Mass Index and Clinical Outcomes Stratified by Age in Patients Undergoing Maintenance Hemodialysis
Source: Hemodial Int. 2026 May 15;30(3):624–34. doi: 10.1111/hdi.70081 (PMC13350522; doi:10.1111/hdi.70081)
Supplement: Supplementary file 1 — Table S1: Medication types and health insurance review and assessment service codes. Table S2: Clinical characteristics according to body mass index in patients aged ≥ 85 years. [file HDI-30-624-s001.docx]

**Table S1. Medication types and health insurance review and assessment service codes**

| **Medications** | **Codes** |
| --- | --- |
| **Alacepril** | 104201ATB, 104202ATB |
| **Benazepril** | 114701ATB |
| **Captopril** | 122901ATB, 122902ATB, 122903ATB |
| **Cilazapril** | 133001ATB, 133002ATB, 133003ATB |
| **Enalapril** | 151601ATB, 151603ATB |
| **Fosinopril** | 163501ATB, 163502ATB |
| **Imidapril** | 173401ATB, 173402ATB |
| **Moexipril** | 196801ATB, 196802ATB |
| **Lisinopril** | 184501ATB |
| **Perindopril** | 211301ATB, 211302ATB, 501601ATB, 501602ATB |
| **Quinapril** | 221901ATB, |
| **Ramipril** | 222401ATB, 222402ATB, 222404ATB |
| **Zofenopril** | 510401ATB, 510402ATB, 510403ATB |
| **Temocapril** | 235002ATB |
| **Delapril** | 140901ATB, 140902ATB |
| **Captopril + Hydrochlorothiazide** | 262200ATB, 262300ATB |
| **Enalapril + Hydrochlorothiazide** | 440300ATB, 453700ATB, 453600ATB |
| **Ramipril + Felodipine** | 447100ATB, 447200ATB |
| **Ramipril + Hydrochlorothiazide** | 448600ATB, 448700ATB |
| **Perindopril + indapamide** | 556200ATB |
| **Lisinopril + Hydrochlorothiazide** | 499200ATB, 499300ATB |
| **Moexipril + Hydrochlorothiazide** | 440800ATB, 497900ATB |
| **Enalapril + nitrendipine** | 466000ATB |
| **Candesartan** | 122601ATB, 122602ATB, 122603ATB |
| **Irbesartan** | 177301ATB, 177303ATB |
| **Losartan** | 185701ATB, 185702ATB |
| **Valsartan** | 247101ATB, 247102ATB, 247103ATB, 247104ATB |
| **Fimasartan** | 515201ATB, 515202ATB, 515203ATB |
| **Azilsartan** | 662401ATB, 662402ATB, 662403ATB |
| **Telmisartan** | 378801ATB, 378802ATB |
| **Eprosartan** | 429201ATB |
| **Olmesartan** | 468501ATB, 468502ATB, 468503ATB, 520901ATB, 520902ATB |
| **Valsartan + Amlodipine** | 492800ATB, 492900ATB, 495800ATB, 522600ABTB, 522700ABTB, 522800ABTB, 522900ABTB, 523000ATB, 523100ATB, 523200ATB, 523300ATB, 523400ATB |
| **Valsartan + Lercanidipne** | 522200ATB. 522300ATB. 522400ATB |
| **Valsartan + Pitavastatin** | 634900ATB, 635000ATB, 635100ATB, 635200ATB |
| **Valsartan + Sacubitril** | 651401ATB, 651402ATB, 651403ATB |
| **Valsartan + Rosuvastatin** | 629700ATB, 629800ATB, 525000ATB, 525100ATB, 525200ATB, 525300ATB, |
| **Valsartan + Hydrochlorothiazide** | 356400ATB, 442600ATB |
| **Olmesaetan + Amlodipine** | 500500ATB, 500600ATB, 547500ATB. 547600ATB, 547700ATB, 547800ATB, 547900ATB, 548000ATB, 582200ATB, 582400ATB, 629400ATB, 629500ATB, 629600ATB, 631300ATB, 632800ATB, 632900ATB, 633000ATB |
| **Olmesartan + Hydrochlorothiazide** | 513600ATB |
| **Olmesartan + Hydrochlorothiazide + Amlodipine** | 519700ATB, 519800ATB, 519900ATB, 520000ATB, 520100ATB |
| **Olmesartan + Rosuvastatin** | 653200ATB, 644100ATB, 644200ATB, 526300ATB, 526400ATB, 526500ATB, 526900ATB |
| **Telmisartan + Hydrochlorothiazide** | 502600ATB, 443200ATB, 443300ATB |
| **Telmisartan + Rosuvastatin** | 629900ATB, 630000ATB, 630100ATB, 630200ATB, 631600ATB, 631700ATB |
| **Telmisartan + Amlodipine** | 511500ATB, 511600ATB, 511700ATB, 521200ATB, 521300ATB, 521400ATB, 623100ATB, 644800ATB |
| **Telmisartan+ Hydrochlorothiazide + Amlodipine** | 663500ATB, 663600ATB, 663700ATB, 663800ATB |
| **Telmisartan + Rosuvastatin + Amlodipine** | 671700ATB, 671600ATB, 671500ATB, 671400ATB, 671300ATB, 671200ATB, |
| **Losartan + Hydrochlorothiazide** | 262500ATB, 378900ATB, 486900ATB |
| **Losartan + Amlodipine** | 502700ATB, 503000ATB, 513900ATB, 637400ATB, 637500ATB, 637600ATB |
| **Losa+rsvt+ Amlodipine** | 663900ATB, 664000ATB, 664100ATB, 664200ATB, 664300ATB, 664400ATB, |
| **Losa+chlor+ Amlodipine** | 662800ATB, 662900ATB, 663000ATB |
| **Fimasartan + Hydrochlorothiazide** | 522000ATB, 526800ATB |
| **Fimasartan + Amlodipine** | 651900ATB, 652000ATB, 652100ATB, 652700ATB, 651900ATB |
| **Fimasartan + Rosuvastatin** | 654600ATB, 654700ATB, 654800ATB, 654900ATB, 655000ATB |
| **Candesartan + Hydrochlorothiazide** | 423700ATB |
| **Candesartan + Amlodipine** | 652900ATB, 653000ATB, 653100ATB, 652900ATB, 652900ATB |
| **Candesartan + Rosuvastatin** | 673700ATB, 661800ATB, 661900ATB, 662000ATB, 662100ATB |
| **Irbesartan + Hydrochlorothiazide** | 385700ATB, 385800ATB |
| **Irbesartan + Atorvastatin** | 527000ATB, 527100ATB, 524000ATB, 524100ATB |
| **Azilsartan + Chlorthalidone** | 673500ATB, 673600ATB |
| **Eprosartan + Hydrochlorothiazide** | 460500ATB |
| **Amlodipine** | 495901ATB, 459802ACH, 483201ATB, 486501ATB, 107601ATB, 107601ATD, 459801ACH, 459801ATB, 459901ATB, 464601ATB, 470801ATB, 476201ATB, 479701ATB, 483202ATB, 486502ATB, 107602ATB, 107602ATD, 470802ATB |
| **Amlodipine + Atorvastatin** | 614500ATB, 472300ATB, 472400ATB, 472500ATB, 518900ATB |
| **Amlodipine + Rosuvastatin** | 673900ATB, 674000ATB, 674100ATB |
| **Amosulalol** | 107901ATB, 107902ATB |
| **Arotinolol** | 110202ATB, 110201ATB |
| **Atenolol** | 483102ATB, 111402ATB, 483101ATB, 111403ATB, 111401ATB |
| **Atenolol + Chlorthalidone** | 262100ATB, 460200ATB |
| **Barnidipine** | 114003ACH, 114001ACH, 114002ACH |
| **Benidipine** | 115101ATB, 115102ATB, 115104ATB, 115103ATB |
| **Betaxolol** | 116801ATB, 116803ATB |
| **Bevantolol** | 117002ATB, 117001ATB |
| **Bisoprolol** | 117904ATB, 117903ATB, 117902ATB, 117901ATB |
| **Bisoprolol + Hydrochlorothiazide** | 469800ATB, 470000ATB, 469900ATB |
| **Carteolol** | 124801ATB |
| **Carvedilol** | 125005ATB, 125003ATB, 662201ATB, 125008ACR, 125001ATB, 662202ATB, 125007ACR, 125002ATB, 125006ACR, 125004ACR |
| **Celiprolol** | 129101ATB |
| **Cilnidipine** | 133102ATB, 133101ATB |
| **Clonidine** | 136505ATR |
| **Diltiazem** | 145706ATB, 145707ACR, 145707ATR, 145703ACR, 145706ATR, 145707ATB |
| **Doxazocin** | 149101ATB, 149102ATB, 149104ATR, 149103ATB |
| **Efonidipine** | 441202ATB, 441201ATB |
| **Felodipine** | 157503ATR, 157501ATR |
| **Felodipine + Metoprolol** | 262400ATR |
| **Hydralazine** | 170701ATB |
| **Lacidipine** | 180301ATB, 180302ATB, 180303ATB |
| **Lercanidipine** | 182001ATB, 182002ATB |
| **Manidipine** | 188001ATB, 188002ATB |
| **Metoprolol** | 194003ATR, 193802ATB, 262400ATR |
| **Metoprolol + Hydrochlorothiazide** | 262600ATB |
| **Metoprolol + Felodipine** | 262400ATR |
| **Minoxidil** | 196102ATB |
| **Nadolol** | 198301ATB |
| **Nebivolol** | 489501ATB, 489502ATB, 489503ATB |
| **Nicardipine** | 201003ACR, 201002ATB |
| **Nifedipine** | 201407ACS, 201405ATR, 528201ATR, 201409ATR, 528202ATR, 201401ACS, 201401ATB, 201408ATR |
| **Nimodipine** | 201901ATB, 356202ATR, 356203ATR, 356201ATB, 356202ATB |
| **Nisoldipine** | 356202ATR |
| **Propranolol** | 219901ATB, 219904ATB, 219906ACR, 219905ACR |
| **Terazosin** | 235501ATB, 235502ATB, 235503ATB, 616501ATB |
| **Verapamil** | 247606ATB, 247607ATB, 247603ATR, 247605ATR, 247601ACR |
| **Atorvastatin + Ezetimibe** | 633800ATB, 633900ATB, 634800ATB |
| **Pitavastatin + Fenofibrate** | 679300ACH |
| **Rosuvastatin + Ezetimibe** | 640700ATB, 640800ATB, 640900ATB |
| **Aspirin** | 110701ATB, 110702ATB, 110801ATB, 110802ATB, 111001ACE, 111001ATB, 111001ATE, 111002ATE, 111003ACE, 111003ATE |
| **Clopidogrel** | 133201ACR, 133201ATB, 133201ATR, 133202ATB, 133203ATR, 506100ATB |
| **Cilostazol** | 136901ATB, 492501ATB, 495201ATB, 498801ATB, 501501ATB |
| **Ticlopidine** | 498900ATB, 239201ATB, 239202ATB |
| **Aspirin + Bethocarbamol** | 256800ATB |
| **Aspirin + Clopidogrel** | 517900ACH, 517900ACE, 517900ATE, 667500ACE |
| **Aspirin + Dipyridamole** | 489700ACR |
| **Atorvastatin** | 111502ATB, 502202ATB, 633900ATB, 472400ATB, 518900ATB, 524100ATB, 527000ATB, 672000ATR, 672100ATR, 111503ATB, 502203ATB, 634800ATB, 472500ATB, 111504ATB, 502204ATB |
| **Fluvastatin** | 162401ACH, 162402ACH, 162403ATR |
| **Lovastatin** | 185801ATB |
| **Pitavastatin** | 470901ATB, 470902ATB, 470903ATB |
| **Pravastatin** | 216601ATB, 216602ATB, 216603ATB, 216604ATB |
| **Rosuvastatin** | 454001ATB, 454002ATD, 454002ATB, 454003ATB, 454003ATD, 454005ATB |
| **Simvastatin** | 227801ATB, 227802ATB, 227803ATB, 227805ATB, 227806ATB |

**Table S2. Clinical characteristics according to body mass index in patients aged ≥ 85 years**

|  | **Underweight**  **(*n* = 182)** | **Normal**  **(*n* = 940)** | **Overweight**  **(*n* = 169)** | **Obese**  **(*n* = 13)** | ***P*** |
| --- | --- | --- | --- | --- | --- |
| Age (years) | 87.5 ± 2.5 | 87.1 ± 2.4 | 86.8 ± 2.0^ab^ | 87.1 ± 2.3 | 0.044 |
| Sex (male, %) | 70 (39%) | 534 (57%) | 72 (43%) | 4 (31%) | <0.001 |
| Body mass index (kg/m^2^) | 17.3 ± 0.9 | 21.7 ± 1.8^a^ | 26.8 ± 1.3^ab^ | 31.6 ± 2.0^abc^ | <0.001 |
| Hemodialysis vintage (months) | 37 ± 31 | 34 ± 33 | 31 ± 28 | 40 ± 51 | 0.424 |
| Underlying causes of end-stage kidney disease |  |  |  |  | 0.014 |
| Diabetes mellitus | 44 (24%) | 338 (36%) | 76 (45%) | 7 (54%) |  |
| Hypertension | 91 (50%) | 374 (40%) | 62 (37%) | 5 (39%) |  |
| Glomerulonephritis | 12 (6.6%) | 35 (3.7%) | 5 (3.0%) | 0 (%) |  |
| Others | 11 (6.0%) | 66 (7.0%) | 6 (3.6%) | 0 (%) |  |
| Unknown | 24 (13%) | 127 (14%) | 20 (12%) | 1 (7.7%) |  |
| Charlson Comorbidity Index score | 8.6 ± 3.2 | 9.0 ± 3.1 | 9.3 ± 3.3 | 9.2 ± 2.8 | 0.306 |
| Arteriovenous fistula | 132 (73%) | 667 (71%) | 113 (67%) | 10 (77%) | 0.622 |
| Kt/V_urea_ | 1.71 ± 0.33 | 1.58 ± 0.23^a^ | 1.50 ± 0.22^ab^ | 1.56 ± 0.42 | <0.001 |
| Ultrafiltration volume (L/session) | 1.6 ± 0.6 | 1.8 ± 0.8^a^ | 1.8 ± 0.8^a^ | 2.2 ± 1.0^a^ | 0.001 |
| Hemoglobin (g/dL) | 10.5 ± 0.7 | 10.6 ± 0.6 | 10.6 ± 0.7 | 10.5 ± 0.6 | 0.424 |
| Serum albumin (g/dL) | 3.66 ± 0.35 | 3.79 ± 0.32^a^ | 3.77 ± 0.31^a^ | 3.78 ± 0.25 | <0.001 |
| Serum phosphorus (mg/dL) | 4.2 ± 1.0 | 4.2 ± 1.0 | 4.2 ± 0.9 | 4.6 ± 0.9 | 0.519 |
| Serum calcium (mg/dL) | 8.60 ± 0.81 | 8.68 ± 0.63 | 8.75 ± 0.66 | 8.79 ± 0.59 | 0.170 |
| Serum creatinine (mg/dL) | 6.6 ± 1.8 | 7.1 ± 1.9^a^ | 7.1 ± 2.2 | 7.1 ± 2.3 | 0.011 |
| Use of renin–angiotensin system blocker | 116 (64%) | 524 (56%) | 79 (47%) | 3 (23%) | 0.001 |
| Use of aspirin | 55 (30%) | 342 (36%) | 51 (30%) | 4 (31%) | 0.221 |
| Use of clopidogrel | 39 (21%) | 228 (24%) | 43 (25%) | 1 (7.7%) | 0.427 |
| Use of statins | 64 (35%) | 472 (50%) | 96 (57%) | 6 (46%) | <0.001 |
| Myocardial infarction or congestive heart failure | 110 (60%) | 567 (60%) | 103 (61%) | 8 (62%) | 0.999 |

Data were presented as mean ± standard deviation for continuous variables and numbers (percentages) for categorical variables. *P*-values were tested using one-way analysis of variance, followed by Tukey’s post hoc test and Pearson’s χ^2^ test for categorical variables.

^a^*P* < 0.05 vs. underweight group; ^b^*P* <0.05 vs. normal group; ^c^*P* < 0.05 vs. overweight group.
